# Anticancer potential, molecular mechanisms and toxicity of *Euterpe oleracea* extract (açaí): A systematic review

**DOI:** 10.1371/journal.pone.0200101

**Published:** 2018-07-02

**Authors:** Jéssica Alessandra-Perini, Karina Cristina Rodrigues-Baptista, Daniel Escorsim Machado, Luiz Eurico Nasciutti, Jamila Alessandra Perini

**Affiliations:** 1 Morphological Science Program—PCM, Biomedical Sciences Institute, Federal University of Rio de Janeiro, Rio de Janeiro, Rio de Janeiro, Brazil; 2 Research Laboratory of Pharmaceutical Sciences—LAPESF, West Zone State University, Rio de Janeiro, Rio de Janeiro, Brazil; 3 Program of Post-graduation in Public Health and Environment—ENSP, National School of Public Health, Oswald Cruz Foundation, Rio de Janeiro, Rio de Janeiro, Brazil; 4 University Center IBMR, Laureate Universities, Rio de Janeiro, Rio de Janeiro, Brazil; 5 Research Division, National Institute of Traumatology and Orthopedics—INTO, Rio de Janeiro, Rio de Janeiro, Brazil; University of Windsor, CANADA

## Abstract

Cancer is an increasingly frequent malignancy worldwide, and despite the advances in drug development, it is still necessary to develop new plant-derived medicines. *Euterpe oleracea* (açaí) is abundant in South and Central America and has health benefits due to its high levels of phytochemicals, including lignans and polyphenols. The aim of this review was to systematically describe the safety and antitumor effects of açaí in preclinical models using rodents to provide a more comprehensive assessment of açaí for both therapeutic uses and the development of future clinical studies in cancer. Eligible studies were identified using four international databases (PubMed, Medline, Lilacs and SciELO) from their inception date through December 2017. The included studies were analyzed with methodological rigor (QATRS) to enable better quality control for these experimental studies. Sixty publications were identified in the databases, but only 9 articles were eligible: 6 evaluated the pharmacological effects of açaí in animal models of cancer (1 model each of esophageal cancer, urothelial cancer, melanoma and Walker-256 tumor and 2 models of colon cancer), and 3 were toxicological assays using preclinical models with rodents. Overall, 747 animals were analyzed. On a QATRS score scale of 0–20, the quality of the studies ranged from 16 to 20 points. Pulp was the main fraction of açaí administered, and an oral administration route was most common. The açaí dosage administered by gavage ranged from 30 mg/kg to 40,000 mg/kg, and açaí fed in the diet accounted for 2.5% to 5% of the diet. The anticarcinogenic and chemopreventive activities of açaí were observed in all experimental models of cancer and reduced the incidence, tumor cell proliferation, multiplicity and size of the tumors due to the antiinflammatory, antiproliferative and proapoptotic properties of açaí. No genotoxic effects were observed after açaí administration. The results of this review suggest that açaí is safe and can be used as a chemoprotective agent against cancer development. Açaí therapy may be a novel strategy for treating cancer.

## Introduction

The use of natural products as medicines accounts for approximately 30% of the currently available drugs [1], and in some therapeutic areas, the amount of plant-derived medicines reaches 60% [2,3]. Brazil has the greatest amount of biodiversity in the world and plays an important role in the area of natural bioactive compounds by contributing natural products to design new clinical medicines [1,4]. Thus, there has been growing research aimed at establishing the therapeutic potential of natural products against several diseases.

*Euterpe oleracea* Mart. is a member of the family Arecaceae and is a typical palm of the rainforest in the Amazon region, in the states of the northern region of Brazil, including Guianas, Colombia, Ecuador, and Venezuela [5]. The fruit, popularly known as “açaí”, weighs approximately 2 g, and the color of the mature fruit is dark purple [6]. Açaí is a traditional food in many regions of Brazil [7,8], and its consumption has increased significantly over the last several years, not only in Brazil but also in Europe and the USA, where the fruit gained popularity after being promoted as a “super fruit” [9]. Currently, due to the health benefits and therapeutic potential of açaí, locally grown açaí are increasingly exported around the world as energy drinks [6,10], “functional foods” [7,8], cosmetics and pharmaceutical products [9]. Açaí pulp is composed of approximately 48% lipids, 13% protein, 8% amino acids, 25% total sugars and minor compounds such as fiber and vitamins (A, B1, B2, B3, C and E) [8,11,12]. Moreover, it is rich in several phytochemicals, including lignans, phenolic compounds (anthocyanins, proanthocyanidins and other flavonoids) and resveratrol, in low concentrations [8,11,12]. The seeds of açaí possess the highest concentration of polyphenols (28.3%), followed by the whole fruit (25.5%) and the bark (15.7%) [13].

The pharmacological effects of açaí are associated with its chemical composition, particularly the presence of bioactive substances, such as phenolics, flavonoids and anthocyanins [14–17]. To date, açaí has been shown to have pharmacological properties including antiinflammatory, antioxidant, cardioprotective and anticancer activities [1,7–9,18,19]. Furthermore, açaí was not shown to be genotoxic *in vitro* and *in vivo* studies conducted, in cultured human lymphocytes and hepatoma cell lines [20], in rodents [21] and in humans [22].

The aim of this review was to systematically describe the safety and antitumor effects of açaí in preclinical models using rodents, to provide a comprehensive assessment of açaí for therapeutic use. Preclinical studies using rodents were evaluated to investigate whether the current knowledge supports cancer clinical trials with açaí.

## Methods

### Search strategy

A careful literature search was performed to identify publications that studied the use of *E*. *oleracea* extract in experimental animal models of cancer and/or evaluated the safety/toxicity of açaí in animal models. Studies were identified by searching the electronic databases: PubMed, Medline-Bireme, Lilacs and SciELO from their inception date through December 2017 (S1 Table). The search terms were as follows: (“*Euterpe oleracea*” AND cancer treatment) OR (“*Euterpe oleracea*” AND cancer animal model) OR (Açaí AND cancer treatment) OR (Açaí AND cancer animal model) AND (“*Euterpe oleracea*” AND toxicity) OR (Açaí AND toxicity). The search was performed without restrictions on the language or year of publication. Two reviewers (KCRB and JA-P) selected the qualified studies independently by browsing the titles, abstracts or full texts based on the eligibility criteria. The duplicates were removed. The eligible articles were separated for analysis of the study methodology and results (S1 Table). Any disagreements were resolved by discussion with two additional reviewers (DEM and JAP).

### Inclusion and exclusion criteria

Articles were included if the following criteria were met: (1) evaluated the pharmacological effect of açaí in animal models of cancer and/or (2) performed toxicological analyzes after açaí administration in experimental animal models. Articles were excluded if the following criteria were met: (1) were reviews of literature; (2) did not analyze the use of açaí *in vivo*; (3) did not use the order *Rodentia*; (4) did not evaluate the toxicological effects of açaí administration *in vivo*; and (5) used only *in vitro* experimental models.

### Data extraction

Three investigators (KCRB, JA-P and JAP) independently conducted the extraction of details from each study including the following: (1) basic information, including the publication year, the first author's name, the type of animals, the sex, the *in vivo* model and the experimental interventions; (2) basic information about the açaí treatment, including the fraction and origin of *E*. *oleracea*, dose, administration route, posology, diluents and treatment groups; and (3) outcome measures used to evaluate *E*. *oleracea* extract, therapeutic indications (pharmacodynamic), açaí signaling pathways and safety evaluations. When a single publication included studies with animals, posology or types of interventions that were different, these data were extracted and considered as independent experiments. Any disagreements regarding the extracted data were resolved by discussion with an additional reviewer (DEM).

### Quality assessment

For assessment of quality, two independent reviewers (KCRB and JA-P) used a quality rating scale as an animal/tissue research scale (QATRS). The QATRS is a 20-point scaled evaluation chart that was designed based on randomization, blinding, the similarity of the animal/tissue model to human applications, standardization and the reliability of the measurement techniques, management of study withdrawals, and appropriateness of the statistical methods [23]. Any disagreements were resolved by discussion with two additional reviewers (DEM and JAP).

## Results

### Study selection

**Fig 1:**
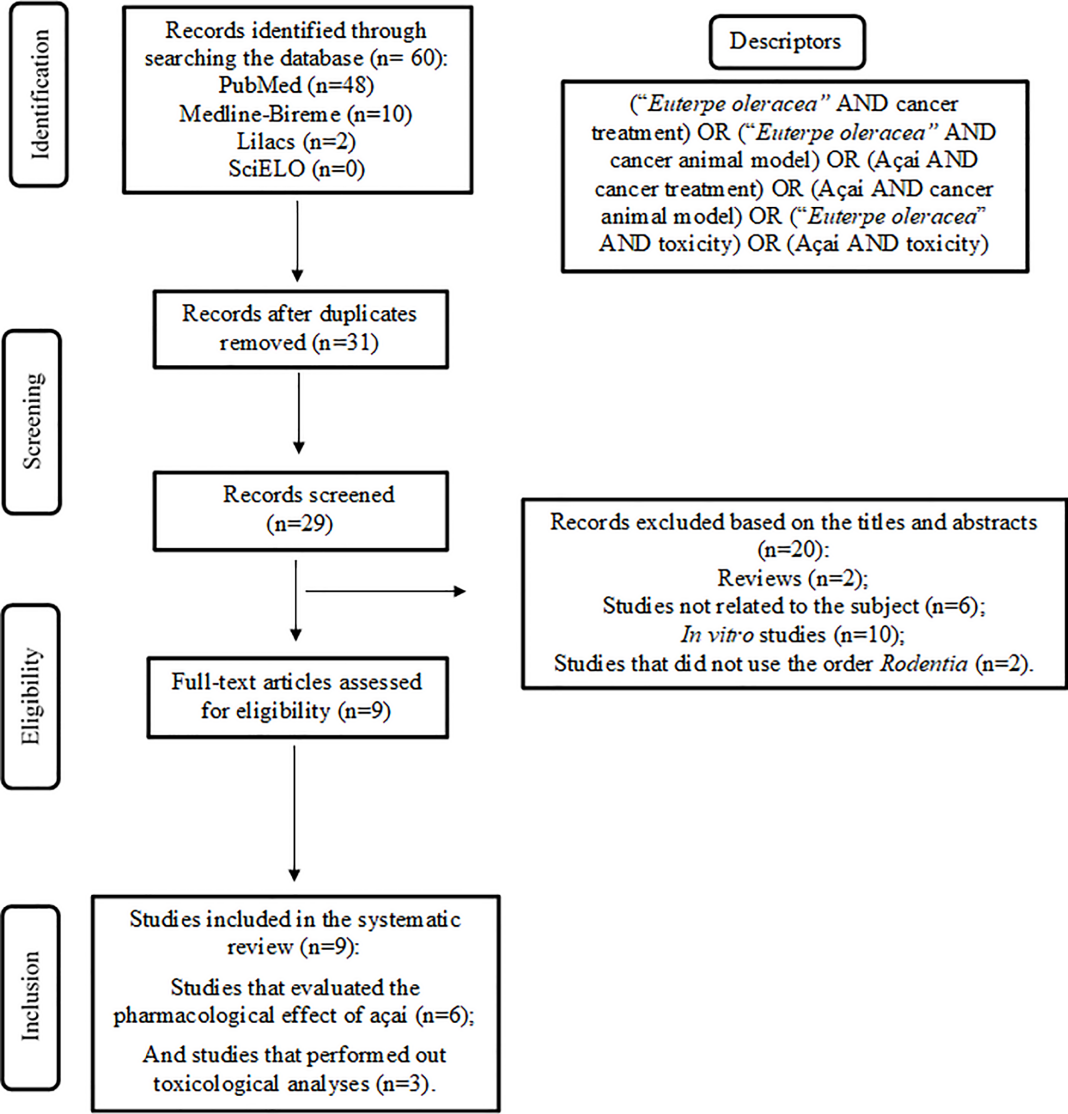
Flowchart of the study selection and inclusion in the review.

A flowchart of the articles that were included in the review is illustrated in Fig 1. A total of 60 publications were identified in the databases; however, 31 were duplicate articles. Among the 29 articles selected, 20 were excluded based on the titles and abstracts because they did not meet the inclusion criteria: 2 were literature reviews [7,24]; 6 did not study açaí in an animal model of cancer and/or did not perform a toxicological analysis [25–30]; 10 were *in vitro* studies [13,20,31–38]; and 2 did not use the order *Rodentia* [21,39]. After reading the full texts 9 articles were included for their critical evaluations of the safety and effectiveness of açaí in animal experimental models [40–48].

### Characteristics of the experimental models

The articles included were analyzed with a critical appraisal tool (QATRS), which allowed for improved quality control of the experimental studies in animal performed independently (see methods). QATRS scores ranged from 0 to 20, and the quality of the studies ranged from 16 to 20 points (Table 1). Among the 9 studies that were included, 6 evaluated the pharmacological effects of açaí in experimental models of cancer, including esophageal [40], urothelial [41], and colon cancer [42,43], and melanoma [44] and Walker-256 tumors [45], and 3 performed toxicological analyses of açaí in experimental models [46–48]. For the interventions used in the experimental models, 4 studies used chemically induced cancer models [40,41,43], 2 used inoculation of tumor cells [44,45], and 3 used models with DNA damage induced by a chemotherapeutic agent [46–48]. The studies involved 2 species and 6 varieties of rodents: C57BL/6 mice [44], F344 rats [40], Wistar rats [42,45,47,48], Swiss mice [41,46], ICR mice [43] and Balb/c mice [48] (Table 1).

**Table 1:** Basic information on the *in vivo* experimental models used to test the effects of *E*. *oleracea*.

Model	Animals	Interventions	References^a^	QATRS
**Cancer**	Male F344 rats	Esophageal carcinogenesis induced by NMBA	Stoner *et al*. 2010	16
	Male Swiss mice	Urothelial carcinogenesis induced by BBN and MNU	Fragoso *et al*. 2012	20
	Male Wistar rats	Colon carcinogenesis induced by DMH	Fragoso *et al*. 2013	20
	Male Wistar rats	Anorexia-cachexia syndrome induced by Walker-256 tumor	Nascimento *et al*. 2016	16
	Male ICR mice	Colon carcinogenesis induced by AOM and DSS	Choi *et al*. 2017	16
	Female C57BL/6 mice	Melanoma induced by transplantation of B16F10 cells	Monge-Fuentes *et al*. 2017	18
**Toxicity**	Male Swiss mice	DNA damage induced by doxorubicin	Ribeiro *et al*. 2010	18
	Male Wistar rats	DNA damage induced by doxorubicin	Marques *et al*. 2016	18
	BALB/c mice	DNA damage induced by cyclophosphamide	Schauss *et al*. 2010^a^	18
	Wistar rats	Acute and subchronic oral toxicity study	Schauss *et al*. 2010^a^	18

AOM = azoxymethane; BBN = N-butyl-N-(4-hydroxybutyl)-nitrosamine; B16F10 = melanoma cell lines; DSS = dextran sulfate sodium; DMH = 1,2-dimethylhydrazine; ICR = International Cancer Research; MNU = N-methyl-N-nitrosourea; NMBA = N-nitrosomethylbenzylamine.

^a^A reference can have more than one model of disease.

### Açaí information

Table 2 shows the basic information about the açaí extract used in the experimental models. The most commonly used açaí fraction was the pulp [40–43,46], followed by the juice [48], oil [44,47] and seeds [45]. Seven studies mentioned the açaí origin, and all of the açaí extracts were from Brazil [40–43,45–47]. The main administration route of açaí was oral; 4 studies administered açaí by gavage [45–48], and 4 studies administered açaí as part of the diet [40–43]. The dosage ranged from 30 mg/kg to 40,000 mg/kg in studies that administered açaí by gavage and was administered as a single dose or as 1 daily dose for 90 consecutive days; in the studies that administered açaí as part of the diet 2.5% to 5% açaí supplementation was provided in the diet for 10 to 35 weeks (Table 2). In addition, Schauss and colleagues used oral and intraperitoneal administration of açaí at a dose of 0.1mg/0.15mL (daily dose during 7 consecutive days) to assess the possible genotoxic effects of açaí using BALB/c mice [48], and Monge-Fuentes and colleagues used 50 mg/mL of açaí administered intratumorally in an experimental model of melanoma [44]. The results regarding the therapeutic indications, effects and safety of açaí in experimental models are summarized in Table 3.

**Table 2:** Basic information regarding the *E*. *oleracea* extract used in the *in vivo* experimental models.

Fraction	Origin of açaí	Dosing	Diluent and placebo	Administration	Posology	Reference
**Juice**^**b**^	Not mentioned	0.1 mg/0.15mL	Saline	Oral (gavage) and IP	1 daily dose over 7 days	Schauss *et al*. 2010^a^
	Not mentioned	5,000 and 20,000 mg/kg	Not mentioned	Oral (gavage)	Single dose	Schauss *et al*. 2010^a^
	Not mentioned	10,000; 20,000 and 40,000 mg/kg	Saline	Oral (gavage)	1 daily dose over 90 days	Schauss *et al*. 2010^a^
**Oil**	Brazil (Amapá)	30, 100 and 300 mg/kg	Tween	Oral (gavage)	1 daily dose over 14 days	Marques *et al*. 2016
	Not mentioned	50 mg/mL	PBS	Intratumoral	Five applications within 15 days (1, 4, 7, 10 and 13 days)	Monge-Fuentes *et al*. 2017
**Pulp**	Brazil	5%	AIN diet	Oral (diet)	35 weeks	Stoner *et al*. 2010
	Brazil (SP)	3,330; 10,000 and 16,670 mg/kg	Saline	Oral (gavage)	Single dose	Ribeiro *et al*. 2010^a^
	Brazil (SP)	3,330; 10,000 and 16,670 mg/kg	Distilled water	Oral (gavage)	1 daily dose over 14 days	Ribeiro *et al*. 2010^a^
	Brazil (Pará)	2.5% and 5%	Standard diet	Oral (diet)	10 weeks	Fragoso *et al*. 2012 and 2013^a^
	Brazil (Pará)	5%	Standard diet	Oral (diet)	20 weeks	Fragoso *et al*. 2013^a^
	Brazil (Pará)	2.5% and 5%	Diet formulated^c^	Oral (diet)	14 weeks	Choi *et al*. 2017
**Seed**	Brazil	100 and 200 mg/mL	Ethanol-water	Oral (gavage)	1 daily dose over 14 days	Nascimento *et al*. 2016

AIN = American Institute of Nutrition; IP = intraperitoneal; SP = São Paulo; PBS = Phosphate buffered saline.

^a^ A reference can have different methods of administration of açaí.

^b^Juice of MonaVie Active® = In addition to açaí, contains lesser amounts of 19 fruits and berries.

^c^A cereal-based commercial diet for mice formulated by the Orient Bio Group (Seongnam, Korea).

**Table 3:** Results of cancer treatments and safety evaluations of *E*. *oleracea* extract in animal models.

References	Therapeutic indication	Action of açaí	Unchanged parameters	Effects of açaí
Stoner *et al*. 2010	Chemopreventive	↓ incidence, multiplicity and inflammatory cytokines; ↑ serum antioxidant capacity and IFNγ	Body weight, food consumption, pro and antiinflammatory	Inhibits esophageal tumorigenesis progression
Fragoso *et al*. 2012	Chemopreventive (anticarcinogenic)	↓ incidence, multiplicity, tumor cell proliferation, urothelial preneoplastic lesions, p63 and PCNA expression and DNA damage	Body weight, food consumption, bladder and kidney weight, kidney biochemical markers, cytoplasmatic and nuclear β-catenin expression	Inhibits urothelial bladder carcinogenesis
Fragoso *et al*. 2013	Chemopreventive	↓ invasiveness, multiplicity and growth of tumor, cell proliferation and cleaved caspase-3, number of aberrant crypts	Body weight, food consumption, β-catenin expression and toxicity	Inhibits colon carcinogenesis
Nascimento *et al*. 2016	Anticarcinogenic	↓ tumor, muscle total protein; ↑ oxidative stress in cerebral cortex	Liver protein, oxidative stress in muscle and liver	Reduces Walker-256 tumor
Choi *et al*. 2017	Anticarcinogenic	↓ incidence, multiplicity and tumor, cell proliferation, proinflammatory cytokines and COX-2; ↑ cleaved-caspase-3 expression.	Not mentioned	Inhibits colon carcinogenesis
Monge-Fuentes *et al*. 2017	Anticarcinogenic (Photodynamic)	↓ tumor, liver and spleen weight; ↑ necrosis; Differences in body weight	Toxicity of the kidneys and lungs	Reduces melanoma carcinogenesis (photosensitizer)
Ribeiro *et al*. 2010	Protective effects	↓ MNPCE and DXR-induced genotoxicity in bone marrow or liver and kidney cells	PCE, DNA damage and genotoxic effects	Reduction in DNA damage induced by DXR
Schauss *et al*. 2010	Not mentioned	Not mentioned	Body weight, food consumption, mortality, organ weights, ophthalmology, urinalysis, hematological and biochemical parameters, and genotoxicity	Negative mutagenic effects
Marques *et al*. 2016	Not mentioned	↑ cell viability	DNA damage, clastogenic and aneugenic effect	Negative genotoxicity effects

COX-2 = cyclooxygenase 2; DXR = antitumoral agent doxorubicin; IFNγ = interferon gamma; MNPCE = number of micronucleated peripheral blood polychromatic erythrocytes cells; PCE = peripheral blood polychromatic erythrocytes cells; PCNA = proliferating cell nuclear antigen.

### Safety of açaí

The absence of toxicity of açaí was reported in 6 studies after testing açaí in experimental models [41,42,44,46–48], and no significant differences in animal body weight or food consumption were reported in 4 studies [40–42,48]. DNA damage induced by antitumor medication was evaluated in 3 studies, and no genotoxic effects were observed after açaí administration by gavage [46–48] (Table 3).

Using a micronucleus test and a comet assay, Ribeiro and colleagues reported no differences between the control and açaí groups in bone marrow and peripheral blood cells polychromatic erythrocytes, and in liver and kidney cells, thus demonstrating the absence of genotoxic effects of açaí. In addition, açaí reduced DNA damage induced by doxorubicin (DXR), suggesting a protective role in human health [46]. In a study done by Schauss and colleagues, açaí did not cause mutagenic effects, as demonstrated by a bacterial reverse mutation assay, a chromosomal aberration assay, a mammalian cell mutation assay and an *in vivo* micronucleus study [48]. In the same way, Marques and colleagues evaluated the genotoxic potential of açaí in rat cells. The authors used a comet assay and a micronucleus test and showed that on both cytogenetic tests, no significant genotoxic effects were observed at the three tested dosages of açaí [47].

### Antitumoral effects of açaí

The anticarcinogenic and chemopreventive activities of açaí, as evidenced by reductions in the incidence of tumors, tumor cell proliferation, and multiplicity and size of tumors, were observed in all the experimental models of cancer [40–45] (Table 3).

Stoner and colleagues reported that açaí was effective at inhibiting the progression of esophageal tumorigenesis, reducing the levels of the serum cytokines (IL-5 and IL-8), and increasing serum antioxidant capacity and interferon-gamma (IFNγ) levels [40]. By contrast, the esophageal tumor size and serum levels of IL-1β, IL-4, IL-13 and tumor necrosis factor-alpha (TNF-α) were not significantly affected by adding açaí to the diet for 35 weeks [40].

Fragoso and colleagues reported that açaí was effective at inhibiting urinary bladder carcinogenesis, reducing DNA damage, and reducing the expression of p63 and proliferating cell nuclear antigen (PCNA) [41]. However, altered cytoplasmatic and nuclear β-catenin were not significantly affected by adding açaí to the diet for 10 weeks [41].

Two studies reported that açaí was effective at inhibiting colon carcinogenesis induced by 1,2-dimethylhydrazine (DMH) in Wistar rats [42] and azoxymethane (AOM) with dextran sulfate sodium (DSS) in ICR mice [43]. Nevertheless, the opposite results were observed with regard to cleaved caspase-3 expression after supplementation with 2.5% and 5% of açaí in the diet for 10 [42], 14 [43] or 20 weeks [42]. Despite the discrepancies between these studies, the quality evaluation of the results of the articles showed good quality QATRS (16/20 and 20/20, respectively) [42,43]. Moreover, Choi and colleagues reported that açaí treatment down-regulated myeloperoxidase (MPO) and proinflammatory cytokines (TNF-α, IL-1β and IL-6), inhibited cyclooxygenase 2 (COX-2), PCNA and Bcl-2, and increased Bad and cleaved caspase-3 expression in an experimental model of cancer colon [43].

Monge-Fuentes and colleagues reported that açaí was an effective photosensitizer because it reduced melanoma carcinogenesis by increaseing the necrotic tissue per tumor area after 5 applications of intratumoral açaí during a period of 15 days [44].

Nascimento and colleagues reported an anticarcinogenic effect (tumor diameter and weight) of açaí in anorexia-cachexia syndrome induced by Walker-256 tumors due to the antioxidant activity of açaí after 1 daily dose of açaí over 14 consecutive days [45].

**Fig 2:**
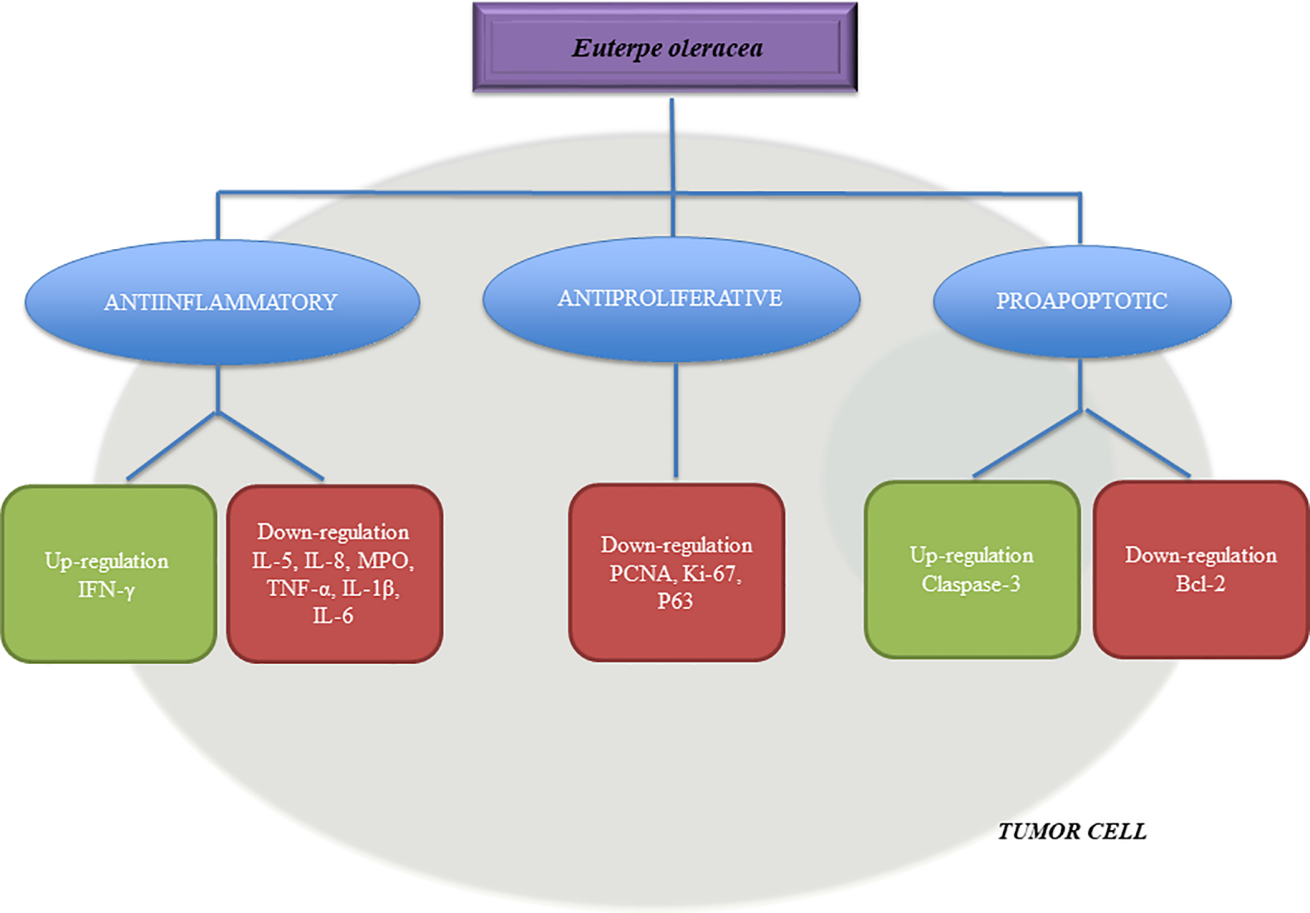
Schematic representation of the effects of açaí on tumor cells. Açaí was shown to have antitumoral functions due its antiinflammatory, antiproliferative and proapoptotic properties.

Finally, based on the results of this review study, we created a schematic representation of the effects of açaí in tumor cells (Fig 2). Açaí showed antitumoral functions due to its antiinflammatory, antiproliferative and proapoptotic properties.

## Discussion

Research on the pharmacological effects of natural products for the treatment of several diseases has significantly increased in the last decades. In this sense, açaí has been marketed as a dietary food supplement because of its health benefits due to its high levels of phytochemicals, including lignans and polyphenols. Studies have demonstrated that açaí has biological effects, such as antioxidant, antiinflammatory, antiproliferative, antinociceptive and antitumorigenic activities [13,16,17,19,49–55].

To the best of our knowledge, 15 clinical trials with açaí was carried [17–19,22,56–66], however none of these studies evaluated the effect of açaí in the cancer treatment. The aim of this review was critically to evaluate the existence of scientific data about the safety and antitumor effects of açaí in preclinical models using rodents, to support cancer clinical trials. The human health benefits of açaí was improvements in antioxidant benefit [17–19,56,61,62,64,65]; cardiovascular health [17,22,56] with beneficial action in hemodynamic [58,60] and metabolic parameters [18,22]; modulation of inflammation [58] and reduction global pain [62]; reduces muscle stress [18,57] and improves effort tolerance in elite athletes [57]; besides to be safe and effective as contrast agent for magnetic resonance imaging [59,63,66]. Due to the important nutritional properties for human benefits and therapeutic potential, the açaí became relevant functional foods.

As far as we know, the present work is the first review to focus on the antitumorigenic and toxicological effects of açaí in preclinical trials using rodents. Overall, nine studies were included in this review [40–48]. Although we conducted a thorough literature search, using four international databases, one limitation is that our conclusions may be narrow due to the lack of availability of published articles and because all of the included studies were published in English. In spite of the small number of studies found, we assessed them with a range of methodological rigor in accordance with the QATRS, which encompasses various aspects that enable better quality control for these experimental studies [23]. A strong point of our review is that all the included studies had good quality as assessed by the QATRS score (all had a score greater than or equal to 16/20). A total of 747 animals of the order *Rodentia* were analyzed. The results indicated that açaí has a chemopreventive effect (anticancer) by inhibiting tumor growth and leads to a reduction in tumor size, suggesting antiproliferative, pro-apoptotic and anti-inflammatory activity [40–45]. In addition, the toxicological studies showed that açaí did not cause DNA damage or genotoxic or mutagenic effects in the evaluated animals, suggesting that it is safe for clinical testing [46–48].

Most of the studies found that açaí significantly decreased tumor incidence or tumorigenic inhibition and prevented DNA damage without causing genotoxic effects when it was administered orally (in the diet or by gavage). These results suggest that the oral route is a good choice for evaluation of the effects of açaí in humans clinical studies since this is an easy and safe route of administration. It should be noted that the significant results found with the oral administration of açaí have also been described in other diseases, such as obesity and hepatic steatosis [67], endometriosis [55], renovascular hypertension [68] and neuropathic pain [53]. The articles included in this review described different doses of açaí that were administered orally by gavage (range 30 mg/kg to 40,000 mg/kg) [45–48]. However, Marques and colleagues observed that at an açaí dose of 300 mg/kg, a few animals showed signs of toxicity (diarrhea and bristling of the hair), which is why they did not test higher doses [47]. Recently, our group reported that a dose of açaí of 200 mg/kg administered by gavage for 30 consecutive days had efficacy in suppressing endometriotic lesions in a Sprague-Dawley rat model without any signs of toxicity [55]. Although considered a benign disease, endometriosis frequently presents with characteristics of malignancy [69]. Therefore, we suggest that an açaí dose of 200 mg/kg is safe for preclinical testing and is a promising novel pharmacological treatment for cancer due to its anticarcinogenic and chemopreventive effects.

With regard to the ability of açaí to inhibit carcinogenesis, and the incidence and multiplicity of tumors in experimental models of cancer using rodents, *in vitro* studies also showed that açaí decreased cell viability, suppressed proliferation and induced apoptosis, suggesting the anticancer and antioxidant activity of açaí against C-6 rat brain glioma cells [49], MCF-7 breast cancer cells [13,38] and colon cancer cells [34]. These results suggest that açaí contains phytochemicals that can be used as natural chemopreventive agents [13,40,42].

A large number of studies have shown the importance of chronic exposure to proinflammatory cytokines in tumorigenesis [70–72]. The results of this review show that açaí acts in the inflammatory processes involved in induced-cancer in animals by decreasing the levels of IL-1β, IL-5, IL-6, IL-8, COX-2, TNF-α and MPO and increasing the levels of IFN-γ [40,43]. An *in vitro* study of polymorphonuclear cells showed a reduction in the IL-8 levels that was associated with the decreasing inflammatory conditions after açaí treatment [64]. Xie and colleagues evaluated flavonoids isolated from açaí pulp and observed a reduction in serum levels, gene expression and protein levels of both the cytokines TNF-α and IL-6 in the resident macrophages cells [73]. Açaí was also able to prevent increases in the levels of IL-1β and TNF-α in the brain tissues of a CCI4 experimental model [27]. In addition, açaí reduced the COX-2 expression and PGE_2_ levels in an experimental model of endometriosis [55] and reduced the MPO levels in a rat renal ischemia/reperfusion model [74].

As a result of this review, it was possible to identify the antiproliferative pathways by which açaí acts by reducing PCNA, Ki-67 and p63 [41–43]. These proteins are involved in tumor development, survival and metastasis of different tumors [75–77]. In addition, the anti-apoptotic proteins Bcl-2 was also reduced after açaí treatment in animals with induced-cancer [43], in agreement with a study of human colon cancer cells in which the proapoptotic activities of polyphenolics from açaí were described [34]. Polyphenolics may regulate distinct steps of the apoptotic process and/or the expression of regulatory proteins, such as the downregulation of Bcl-2 and cleavage of caspase-3 [78,79]. Açaí polyphenolics were previously described to have proapoptotic and antiproliferative activities in leukemia cancer cells through caspase-3 activation [80]. Surprisingly, as a result of this review, it was possible to identify the discrepancies in the levels of cleaved caspase-3 in colon carcinogenesis induced after açaí treatment [41,43]. Choi and colleagues observed that açaí increased the cleaved caspase-3 levels in the supernatants of colon strips [43], but Fragoso and colleagues described the opposite results using immunohistochemical techniques in colon tumor tissues [41].

Another specie from Brazilian *Euterpe*, o *Euterpe edulis*, has been studied because has important nutritional properties for human health. *E*. *edulis* Mart., commonly known as juçara or jussara and açaí-do-sol, is a native tree of the Atlantic Forest and has similar nutritional properties of açaí [81], however açaí has twice of the polyphenols concentration [82]. Recently, a review described 25 articles about the phytochemical characterization and biological activities of juçara [81]. Nevertheless, none of these studies evaluated the effect of *E*. *edulis* in the cancer treatment and two studies described the safety evaluation of *E*. *edulis*, however with controversial results. Barros Freitas et al, 2017 showed juçara prevent the oxidative damage resulting from the cafeteria diet and no evidenced signs of lipid peroxidation in renal or in cardiac tissue in Wistar rats [82]. On the other hand, Felzenszwald et al., 2013, demonstreted *E*. *edulis* was able to induce mutagenicity and clastogenic/aneugenic effects in Wistar rats [25].

Toxicity data are decisive for evaluating the safety of natural products for clinical treatment because these data investigate the potential for mutagenicity, genotoxicity, clastogenicity and aneugenicity [83]. The toxicological studies included in this review showed that açaí is non-toxic [42,44,46–48], has no genotoxic or mutagenic effects, and has a protective effect on DNA damage caused by antitumoral agents [46–48]. Similarly, Santos and colleagues showed that antioxidant compounds prevented the induction of DNA damage induced by DXR [84]. On the other hand, açaí showed mutagenic effects when assayed in high concentrations in eukaryotic cells of *Saccharomyces cerevisiae* yeast; however, there is a low mutagenic risk for humans because the tested concentrations were significantly elevated [31]. Since only 3 studies investigated the genetic toxicity of açaí in preclinical trials of rodents [46–48], future research is needed to better understand the efficacy of açaí because its antimutagenic and antioxidant activities may prevent DNA damage and thus improve human health.

## Conclusions

The results of this review suggest that açaí is safe and can be used as a chemoprotective agent against cancer by exhibiting antiinflammatory, antioxidant, antiproliferative, and proapoptotic properties. Further studies on the functional relevance of açaí are necessary to build a database that can be used in future clinical investigations aimed at discovering antitumor agents.

## Supporting information

S1 Table Complete search on açaí in databases. (PDF)

S2 Table PRISMA checklist. (PDF)
