# Supplementary material for: Anticancer potential, molecular mechanisms and toxicity of Euterpe oleracea extract (açaí): A systematic review
Source: PLoS One. 2018 Jul 2;13(7):e0200101. doi: 10.1371/journal.pone.0200101 (PMC6028114; doi:10.1371/journal.pone.0200101)
Supplement: S1 Table — (PDF) [file pone.0200101.s001.pdf]

Complete Search on açaí in databases

Full Search				
	**Pubmed**	**Medline-Bireme**	**SciELO**	**Lilacs**
1	Brasil A, 2017	Brasil A, 2017		Silva DF, 2013
2	Brasil A, 2017	Brito C, 2016		Silva DF, 2013
3	Brito C, 2017	Brito C, 2017		
4	Brito C, 2017	Caiado RR, 2017		
5	Brito C, 2016	Dias MM, 2014		
6	Brito C, 2016	Felzenszwalb I, 2013		
7	Caiado RR, 2017	Kowar M, 2015		
8	Caiado RR, 2017	Marques ES, 2016		
9	Choi YJ, 2017	Schauss AG, 2010		
10	de Souza Machado F, 2015	Spada PD, 2008		
11	Dias MM, 2014			
12	Dias MM, 2014			
13	Felzenszwalb I, 2013			
14	Felzenszwalb I, 2013			
15	Fragoso MF, 2012			
16	Fragoso MF, 2013			
17	Fragoso MF, 2013			
18	Fragoso MF, 2013			
19	Fragoso MF, 2013			
20	Freitas DDS, 2017			
21	Freitas DDS, 2017			
22	Kim YS, 2013			
23	Kim YS, 2013			
24	Kinghorn AD, 2011			
25	Kinghorn AD, 2011			
26	Leba LJ, 2016			
27	Machado AK, 2016			
28	Machado AK, 2016			
29	Marques ES, 2017			
30	Marques ES, 2017			
31	Marques ES, 2016			
32	Marques ES, 2016			
33	Monge Fuentes V, 2017			
34	Monge Fuentes V, 2017			
35	Nascimento VH, 2016			
36	Nascimento VH, 2016			
37	Nascimento VH, 2016			
38	Nascimento VH, 2016			
39	Ribeiro JC, 2010			
40	Ribeiro JC, 2010			
41	Schauss AG, 2010			
42	Schauss AG, 2010			
43	Schreckinger ME, 2010			

44	Silva DF, 2014			
45	Silva DF, 2014			
46	Stoner GD, 2010			
47	Vrailas MA, 2012			
48	Wong DY, 2013			

Duplicates Articles				
	**Pubmed**	**Medline-Bireme**	**SciELO**	**Lilacs**
1	Brasil A, 2017	Brasil A, 2017		Silva DF, 2013
2	Brito C, 2017	Brito C, 2016		
3	Brito C, 2016	Brito C, 2017		
4	Caiado RR, 2017	Caiado RR, 2017		
5	Dias MM, 2014	Dias MM, 2014		
6	Felzenszwalb I, 2013	Felzenszwalb I, 2013		
7	Fragoso MF, 2013	Marques ES, 2016		
8	Fragoso MF, 2013	Schauss AG, 2010		
9	Fragoso MF, 2013			
10	Freitas DDS, 2017			
11	Kim YS, 2013			
12	Kinghorn AD, 2011			
13	Machado AK, 2016			
14	Marques ES, 2017			
15	Marques ES, 2016			
16	Monge Fuentes V, 2017			
17	Nascimento VH, 2016			
18	Nascimento VH, 2016			
19	Nascimento VH, 2016			
20	Ribeiro JC, 2010			
21	Schauss AG, 2010			
22	Silva DF, 2014			

Articles after duplicates removed				
	**Pubmed**	**Medline-Bireme**	**SciELO**	**Lilacs**
1	Brasil A, 2017	Kowar M, 2015		Silva DF, 2013
2	Brito C, 2017	Spada PD, 2008		
3	Brito C, 2016			
4	Caiado RR, 2017			
5	Choi YJ, 2017			
6	de Souza Machado F, 2015			
7	Dias MM, 2014			
8	Felzenszwalb I, 2013			
9	Fragoso MF, 2012			
10	Fragoso MF, 2013			
11	Freitas DDS, 2017			

12	Kim YS, 2013			
13	Kinghorn AD, 2011			
14	Leba LJ, 2016			
15	Machado AK, 2016			
16	Marques ES, 2017			
17	Marques ES, 2016			
18	Monge Fuentes V, 2017			
19	Nascimento VH, 2016			
20	Ribeiro JC, 2010			
21	Schauss AG, 2010			
22	Schreckinger ME, 2010			
23	Silva DF, 2014			
24	Stoner GD, 2010			
25	Vrailas MA, 2012			
26	Wong DY, 2013			

Included Articles		
	**Treatment Animal Model**	**Toxicological Animal Model**
1	Choi YJ, 2017	Ribeiro JC, 2010
2	Fragoso MF, 2012	Schauss AG, 2010
3	Fragoso MF, 2013	Marques ES, 2016
4	Nascimento VH, 2016	
5	Monge Fuentes V, 2017	
6	Stoner GD, 2010	

Excluded Articles				
	**Pubmed**	**Medline-Bireme**	**SciELO**	**Lilacs**
1	Brasil A, 2017	Kowar M, 2015		Silva DF, 2013
2	Brito C, 2017	Spada PD, 2008		
3	Brito C, 2016			
4	Caiado RR, 2017			
5	de Souza Machado F, 2015			
6	Dias MM, 2014			
7	Felzenszwalb I, 2013			
8	Freitas DDS, 2017			
9	Kim YS, 2013			
10	Kinghorn AD, 2011			
11	Leba LJ, 2016			
12	Machado AK, 2016			
13	Marques ES, 2017			
14	Schreckinger ME, 2010			
15	Silva DF, 2014			
16	Vrailas MA, 2012			
17	Wong DY, 2013			

Reason for excluded the artciles				
	**Reviews**	**Not related to subject**	***In vitro* studies**	**Do not used the order *Rodentia***
1	Kinghorn AD, 2011	Brasil A, 2017	Brito C, 2017	Caiado RR, 2017
2	Schreckinger ME, 2010	de Souza Machado F, 2015	Brito C, 2016	Vrailas MA, 2012
3		Felzenszwalb I, 2013	Dias MM, 2014	
4		Kim YS, 2013	Freitas DDS, 2017	
5		Kowar M, 2015	Machado AK, 2016	
6		Leba LJ, 2016	Marques ES, 2017	
7			Wong DY, 2013	
8			Silva DF, 2014	
9			Silva DF, 2013	
10			Spada PD, 2008	
